# Conservative management of razor blade ingestion

**DOI:** 10.1093/gastro/gou002

**Published:** 2014-02-13

**Authors:** Mazen Albeldawi, Sigurbjorn Birgisson

**Affiliations:** Gastroenterology and Hepatology, Cleveland Clinic, Cleveland, OH 44195, USA

**Keywords:** Foreign body ingestion, endoscopy, razor blades

## Abstract

A 30-year-old woman presented to the emergency department complaining of abdominal discomfort. An abdominal radiograph was done, revealing ten rectangular razor blades measuring 5 × 2 cm. The patient was taken to the operating room and a flexible esophago-gastroduodenoscopy was performed. Attempts at retrieval, using both a gastric overtube and an inverted hood, were unsuccessful due to the shape and size of the blades. She was transferred to a regular medical floor and managed conservatively with serial abdominal radiographs. Over the next week, she passed the razor blades transanally without further event—all were still wrapped in paper and chewing gum—and was cleared to be discharged home.

## CASE PRESENTATON

A 30-year-old woman presented to the emergency department, complaining of abdominal discomfort. She had a past medical history of borderline personality disorder, atypical schizo-affective disorder, a long history of self-mutilating behavior and numerous suicide attempts. Given her history of previous foreign body ingestion an abdominal radiograph (Panel A) was carried out, revealing ten rectangular razor blades, each measuring 5 x 2 cm. They were closely grouped at the dependent portion of the body of the stomach, without evidence of pneumoperitoneum or pneumomediastinum. The patient was taken to the operating room and a flexible esophago-gastroduodenoscopy was performed. The esophagus appeared grossly normal and the stomach revealed multiple razor blades, which were wrapped in paper and chewing gum. Attempts at retrieval, using both a gastric overtube and an inverted hood, were unsuccessful due to the shape and size of the blades. Given the patient’s history of numerous exploratory laparotomies for foreign body ingestions with lysis of adhesions, surgical retrieval was deferred. The patient was informed of potential complications, including perforation or obstruction, and the need for surgical intervention if these adverse events were to occur.

## DISCUSSION

All overtubes have an inner diameter greater than an endoscope, providing a conduit for passage of the device into the digestive tract. Endoscopic overtubes vary in length (23–135 cm) and caliber (outer diameters 14.4–21 mm) and are intended to protect the gastrointestinal (GI) mucosa from trauma and limit the risk of aspiration [1]. The majority of ingested foreign objects will pass spontaneously, without the need for intervention [2]. However, the ingestion of sharp and pointed objects increases the risk of perforation as much as 35% [2]. Impaction, perforation or obstruction often occurs at GI angulations or narrowing [3]. However, once through the esophagus most foreign bodies, including sharp objects, pass uneventfully [4]. Current guidelines recommend that surgical intervention be considered for objects that fail to progress after 3 days [4].

## CONCLUSION

The patient was transferred to a regular medical floor and managed conservatively with serial abdominal radiographs (Panels B and C). Over the next week, she passed the razor blades transanally without further event, all still wrapped in paper and chewing gum, and was cleared to be discharged home with outpatient follow-up with psychiatry. Since being discharged, the patient has not been re-admitted to our institution for further episodes of foreign body ingestion.

**Conflict of interest:** none declared.
